# Characterizing the *Wolbachia* infection in field-collected Culicidae mosquitoes from Hainan Province, China

**DOI:** 10.1186/s13071-023-05719-y

**Published:** 2023-04-14

**Authors:** Yiji Li, Yingbo Sun, Jiaquan Zou, Daibin Zhong, Rui Liu, Chuanlong Zhu, Wenting Li, Yanhe Zhou, Liwang Cui, Guofa Zhou, Gang Lu, Tingting Li

**Affiliations:** 1 Key Laboratory of Tropical Translational Medicine of Ministry of Education, Hainan Medical University, Haikou, 571199 China; 2 Tropical Diseases Research Center, Department of Pathogen Biology, Hainan Medical University, Haikou, 571199 China; 3 Hainan Medical University-The University of Hong Kong Joint Laboratory of Tropical Infectious Diseases, Hainan Medical University, Haikou, 571199 China; 4 Program in Public Health, College of Health Sciences, University of California at Irvine, Irvine, CA 92617 USA; 5 Department of Infectious and Tropical Diseases, The Second Affiliated Hospital of Hainan Medical University, Haikou, 570311 People’s Republic of China; 6 Guangzhou Women and Children’s Medical Center, Guangdong Provincial Clinical Research Center for Child Health, Guangzhou Medical University, Guangzhou, 510623 China; 7 NHC Key Laboratory of Tropical Disease Control, Hainan Medical University, Haikou, 571199 Hainan China; 8 Department of Internal Medicine, Morsani College of Medicine, University of South Florida, Tampa, FL 33612 USA; 9 The Second Affiliated Hospital, Hainan Medical University, Haikou, 570311 China; 10 Academician Workstation of Hainan Province, Hainan Medical University, Haikou, 571199 People’s Republic of China

**Keywords:** *Wolbachia*, Mosquito, Species diversity, *Wsp*, *FtsZ*, *16S* rRNA, Genetic diversity, Phylogeny

## Abstract

**Background:**

Mosquitoes are vectors of many pathogens, such as malaria, dengue virus, yellow fever virus, filaria and Japanese encephalitis virus. *Wolbachia* are capable of inducing a wide range of reproductive abnormalities in their hosts, such as cytoplasmic incompatibility. *Wolbachia* has been proposed as a tool to modify mosquitoes that are resistant to pathogen infection as an alternative vector control strategy. This study aimed to determine natural *Wolbachia* infections in different mosquito species across Hainan Province, China.

**Methods:**

Adult mosquitoes were collected using light traps, human landing catches and aspirators in five areas in Hainan Province from May 2020 to November 2021. Species were identified based on morphological characteristics, species-specific PCR and DNA barcoding of *cox*1 assays. Molecular classification of species and phylogenetic analyses of *Wolbachia* infections were conducted based on the sequences from PCR products of *cox*1, *wsp*, *16S* rRNA and *FtsZ* gene segments.

**Results:**

A total of 413 female adult mosquitoes representing 15 species were identified molecularly and analyzed. Four mosquito species (*Aedes albopictus*, *Culex quinquefasciatus*, *Armigeres subalbatus* and *Culex gelidus*) were positive for *Wolbachia* infection. The overall *Wolbachia* infection rate for all mosquitoes tested in this study was 36.1% but varied among species. *Wolbachia* types A, B and mixed infections of A × B were detected in *Ae. albopictus* mosquitoes. A total of five *wsp* haplotypes, six *FtsZ* haplotypes and six *16S* rRNA haplotypes were detected from *Wolbachia* infections. Phylogenetic tree analysis of *wsp* sequences classified them into three groups (type A, B and C) of *Wolbachia* strains compared to two groups each for *FtsZ* and *16S* rRNA sequences. A novel type C *Wolbachia* strain was detected in *Cx. gelidus* by both single locus *wsp* gene and the combination of three genes.

**Conclusion:**

Our study revealed the prevalence and distribution of *Wolbachia* in mosquitoes from Hainan Province, China. Knowledge of the prevalence and diversity of *Wolbachia* strains in local mosquito populations will provide part of the baseline information required for current and future *Wolbachia*-based vector control approaches to be conducted in Hainan Province.

**Graphical Abstract:**

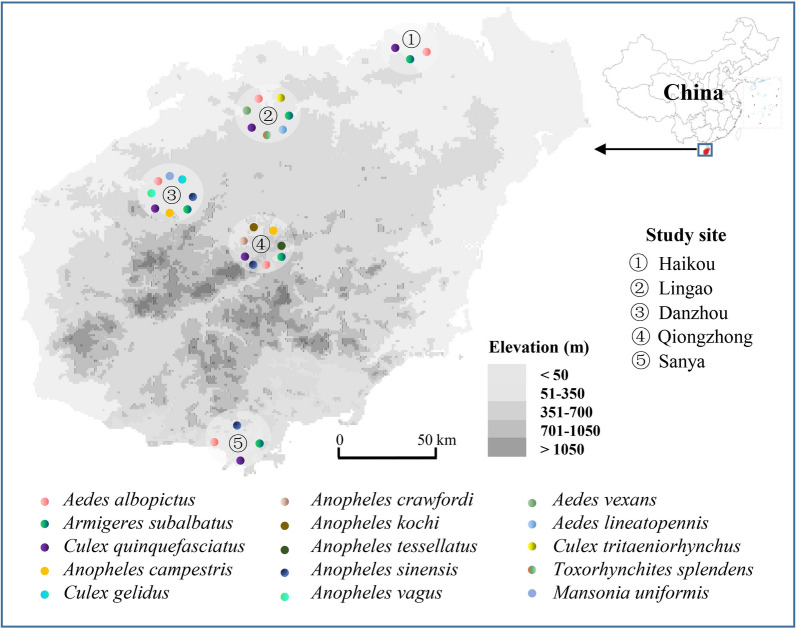

**Supplementary Information:**

The online version contains supplementary material available at 10.1186/s13071-023-05719-y.

## Background

*Wolbachia* belongs to the family Ehrlichiaceae in the order Rickettsiales. It is a group of endosymbiotic bacteria which is maternally inherited and found in many species of arthropods and nematodes [1, 2]. It is estimated that *Wolbachia* naturally infects as many as 25–70% of insect species [3–5], including a large range of mosquito vector species that are responsible for transmitting diseases in humans such as malaria, dengue, yellow fever, filariasis and Japanese encephalitis [1, 6, 7]. *Wolbachia* can induce reproductive manipulation phenotypes, including parthenogenesis, feminization, cytoplasmic incompatibility and male-killing, which increase the endosymbiont’s reproductive success [8–10].

Traditional insecticide-based vector control measures are widely used for transmission reduction and disease prevention [11]. Due to widespread mosquito resistance to chemical insecticides [12, 13], new viable alternatives are vital for vector and pathogen transmission control. *Wolbachia*-based biological control is one of those novel alternatives [14]. It is an ecologically friendly and potentially cost-effective method for the prevention and control of many arboviral infections such as dengue and Zika viruses [15]. In *Aedes* mosquitoes, *Wolbachia* can induce cytoplasmic incompatibility (CI), i.e. when *Wolbachia*-infected male mosquitoes mate with uninfected females, viable offspring are not produced. This serves as the basis for the suppression of field *Aedes* mosquito population, i.e. mass-rearing and mass release of *Wolbachia*-infected male mosquitoes to suppress the field *Aedes* mosquito population while preventing dengue virus transmission, the so-called population suppression strategy [14]. Such a mass release has been conducted in serval countries such as China, Singapore, Australia and the USA [17–20]. Another strategy is population replacement followed by suppression, aiming to reduce the natural mosquito population size after the *Wolbachia* infection has been established [14]. Once the *Wolbachia* infection is at a high frequency, host fitness costs can reduce the size of the population by the reduced mosquito survival or fertility [21]. In addition, when a combination of different strains of *Wolbachia* is introduced into *Aedes* mosquito eggs, the dengue virus is unable to replicate in the modified mosquitoes that hatch [22]. These pathogen-blocking effects serve as the principle for direct dengue virus transmission control because the females pass the *Wolbachia* to their offspring; mass release of pathogen-blocking *Wolbachia*-infected female *Aedes* mosquitoes can lead to reduced dengue virus-carrying female *Aedes* mosquitoes [23, 24]. We have to keep in mind that simple natural infection such as mono-*w*AlbA or -*w*AlbB or combined *w*AlbA and *w*AlbB may not be enough to fully prevent arboviral infections [25]. In fact, not all the population replacement programs were successful [26], and choosing the right *Wolbachia* strain is key for the success [14]. All these indicate the importance of research on *Wolbachia* ecology and population genetics.

Although *Wolbachia*-infected mosquitoes have been tested as biocontrol agents in the field in China [16], the presence of naturally occurring endosymbionts such as *Wolbachia* in wild (field-collected) mosquito populations has not been adequately assessed [27–29]. Understanding *Wolbachia* infection prevalence, bacteria strains, infected mosquito species and spatial distribution of infections is essential for developing future vector control and disease prevention strategies.

Hainan Province, the largest island province in the South China Sea, has a tropical climate and is an ideal place for the development and survival of mosquitoes. More than 60 species of mosquitoes were reported in Hainan Province in the 1960s [30], and recent studies reported more than 20 species [31, 32]. Many mosquito-borne diseases, such as malaria, dengue and filariasis, have recently been or still are prevalent in Hainan Province; for example, a dengue fever outbreak occurred there in 2019 [33, 34]. Therefore, from a disease prevention point of view, it would be very useful to understand the prevalence and phylogenetic relationship of *Wolbachia* among different mosquito species.

This study had two research objectives. The first aim was to examine the natural prevalence of *Wolbachia* infections among wild mosquitoes collected from areas with different ecological settings in Hainan Province using *Wolbachia*-specific DNA markers, *Wolbachia* surface protein (*wsp*) and PCR-based molecular approaches. The second aim was to determine the genetic diversity and phylogenetic relationships of *Wolbachia* strains among wild-collected mosquitoes based on *wsp*, *16S rRNA* and cell division protein FtsZ (*FtsZ*) markers.

## Methods

### Study sites and mosquito sampling

**Fig. 1:**
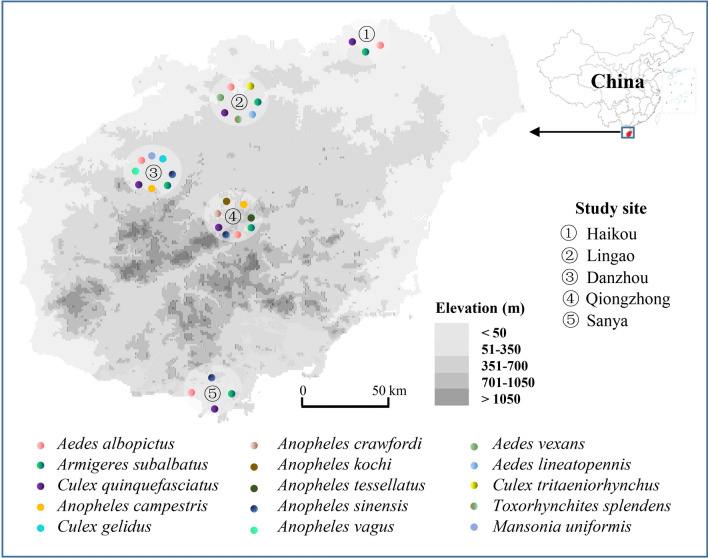
Map of the study sites and mosquito species distribution in Hainan Province, China. Study sites: (1) Haikou, (2) Lingao, (3) Danzhou, (4) Qiongzhong, (5) Sanya

Five study sites with different ecological settings were selected to examine the *Wolbachia* natural infection status in different mosquito species across Hainan Province between May 2020 and November 2021 (Fig. 1). Three methods were deployed to collect the adult mosquito samples: CDC light trap, human landing catch and hand aspirator. Mosquitoes were morphologically identified using taxonomic keys [35]. A subset of 413 female mosquitoes from different species was preserved in ethyl alcohol at − 20 °C for subsequent molecular species identification, *Wolbachia* detection and population genetics analyses.

### DNA extraction and mosquito species identification

Before DNA extraction, all mosquito samples (*n* = 413) were surface sterilized with 75% ethanol for 5 min followed by washing with phosphate-buffered saline (PBS) twice. Genomic DNA was extracted from mosquitoes individually using the method published by Chang et al. [36]. The extracted DNA was run on a 1.0% agarose gel electrophoresis to confirm its presence. Then, extracted DNA was stored at − 20 °C or used immediately for PCR.

For mosquito species identification, mosquitoes were first morphologically divided into *Anopheles*, *Culex*, *Aedes*, *Armigeres* and other species. Molecular identifications of *Anopheles sinensis*, *Culex quinquefasciatus* and *Aedes albopictus* were conducted using species-specific PCR primers (forward: TGTGAACTGCAGGACACATGAA and reverse: AGGGTCAAGGCATACAGAAGGC for *An. sinensis* [37]; forward: CCTTCTTGAATGGCTGTGGCA and reverse: TGGAGCCTCCTCTTCACGG for *Cx. quinquefasciatus* [38]; forward: CACCCGTGTATGTGCGATATTA and reverse: TTGGTCGTTCGGTGGTAAAG for *Ae. albopictus* [39]). For other mosquito species identification, Sanger sequencing was performed to target a fragment of the cytochrome c oxidase subunit I (*cox1*) gene using primers LCO1498 (5ʹ-GGTCAACAAATCATAAAGATATTGG-3ʹ) and HCO2198 (5ʹ-TAAACTTCAGGGTGACCAAAAAATCA-3ʹ) [40]. PCR procedures were performed in reaction mixtures consisting of 12.5 μl of DreamTaq^™^ Green PCR Master Mix (2×) (Thermo Scientific, USA), 1 μl extracted DNA and 1 μl each of 10-μM forward and reverse primers. Double-distilled water was used to top up the reaction mixture to a final volume of 25 μl. PCR amplification of positive and negative controls was also conducted simultaneously. PCR conditions were as follows: 94 °C for 5 min, followed by 35 cycles of 95 °C for 30 s, 53 °C for 45 s and 72 °C for 1 min, with a final elongation step of 72 °C for 10 min.

### PCR identification of *Wolbachia* infections in field-collected mosquitoes

Detection of the *Wolbachia* endosymbiont in mosquitoes was performed using the most commonly used *Wolbachia*-specific DNA marker (*wsp* gene) and PCR-based molecular approaches with forward primer (81F: TGGTCCAATAAGTGATGAAGAAAC) and reverse primer (691R: AAAAATTAAACGCTACTCCA) [41]. To classify *Wolbachia* groups of infected *Ae. albopictus*, further PCR amplification of the *wsp* gene was conducted using wAlbA primers (328F: 5ʹ-CCAGCAGATACTATTGCG-3ʹ and 691R: 5ʹ-AAAAATTAAACG CTACTCCA-3ʹ) for A group and wAlbB primers (183F: 5ʹ-AAGGAACCGAAGTTCATG-3' and 691R: 5'-AAAAATTAAACGCTACTCCA-3') for B group [41]. PCR amplification was performed in a 25-μl reaction volume with 12.5 μl DreamTaq^™^ Green PCR Master Mix (2×) (Thermo Scientific, USA), 0.5 μl each of the forward and reverse primers at 10 μmol/l, 0.5 μl of template DNA and sufficient nuclease-free water to make 25 μl. PCR conditions were as follows: an initial denaturation at 94 °C for 3 min followed by 35 cycles of 94 °C for 30 s, 55 °C for 30 s and 72 °C for 1 min, and a final extension at 72 °C for 5 min. Five microliters of the PCR products was run on 1.5% agarose gel with a DL2000 DNA marker (Zomanbio, Beijing, China) to confirm the PCR amplification. PCR-amplified fragments of 364 bp and 509 bp for wAlbA and wAlbB, respectively, were revealed under UV light after electrophoresis. Sanger sequencing of PCR products was conducted on a subset of PCR-positive samples to confirm *Wolbachia* infections.

### Genetic diversity and phylogenetic relationship of *Wolbachia* strains

To determine the genetic diversity and phylogenetics of naturally infected *Wolbachia* strains in different mosquito species, we conducted DNA sequencing of the three conserved *Wolbachia* genes: *16S* rRNA gene [42–44], *Wolbachia* surface protein (*wsp*) gene [41] and *Wolbachia* cell division protein (*FtsZ*) gene [45]. Primers used are shown in Additional file 1: Table S1. DNA extracted from Haikou adult *Aedes albopictus* (infected with the wAlbA and wAlbB strains of *Wolbachia*) was used as a positive control [46] in addition to no-template controls (NTCs). PCR amplifications were performed in reaction mixtures consisting of 12.5 μl of DreamTaq™ Green PCR Master Mix (2×) (Thermo Scientific, USA), 0.5 μl of extracted DNA and 1 μl each of 10-μM *wsp* forward and reverse primers for *Wolbachia* PCR screens. Double-distilled water was used to top up the reaction mixture to a final volume of 25 μl. PCR conditions were as follows: 94 °C for 5 min, followed by 35 cycles of 95 °C for 30 s, 55 °C for 45 s for *wsp* and *16S* rRNA gene primers or 60 °C for 45 s for *FtsZ* cell cycle gene primers, and 72 °C for 1 min, with a final elongation step of 72 °C for 10 min. Nested PCR amplifying the *16S* rRNA gene was used to detect *Wolbachia* in all mosquito samples. The initial PCR employed *16S Wolbachia*-specific primers (W-Specf: 5ʹ-CATACCTATTCGAAGGGATAG-3ʹ; W-Specr: 5ʹ-AGCTTCGAG TGAAACCAATTC-3ʹ) and was performed in a 25-µl reaction volume using 2 µl DNA [43]. Then, 2 µl of the initial PCR products was amplified in a 25 µl PCR reaction using specific internal primers (16SNF: 5ʹ-GAAGGGATAGGGTCGGTT CG-3ʹ; 16SNR: 5ʹ-CAATTCCCATGGCGTGACG-3ʹ) [42]. All amplicons were separated by gel electrophoresis on 1.5% agarose gel stained with GoodView Nucleic Acid Stain (Sbsbio, Beijing, China) and visualized under an ultraviolet fully automatic digital gel imaging analysis system (Tanon, Shanghai, China). PCR products were submitted to Sangon Biotech (Sangon BiotechCo., Ltd, Shanghai, China) for PCR reaction cleanup, followed by Sanger sequencing to generate both forward and reverse reads, using a 3730XL DNA Analyzer (Applied Biosystems, Waltham, MA, USA).

### Data analysis

The CodonCode Aligner 9.0.2 (CodonCode Corporation, Centerville, MA, USA) was used to check the sequence quality and trim low-quality bases. Ambiguous sequences were omitted from the results. BioEdit Sequence Alignment Editor software [47] was used to align the sequences. All aligned DNA sequences were compared with other sequences available in the GenBank database to determine the percentage identity using BLAST (https://blast.ncbi.nlm.nih.gov/Blast.cgi), and the most similar sequences were downloaded for phylogenetic analysis. Phylogenetic trees were constructed using MEGA version X software [48]. Phylogenetic relationships were inferred using the UPGMA method. Nucleotide sequences generated in this study have been submitted to GenBank (accession numbers OP279050-OP279063, OP367764-OP367777, OP363894-OP363900, OP393144-OP393149 and OP426265-OP426271).

## Results

### Mosquito abundance and diversity at study sites

Overall, 413 female individuals belonging to six genera and 15 species were identified from the five collection sites (Table 1). Among them, 173 (41.9%) belonged to *Anopheles*, 80 (19.4%) to *Culex*, 112 (27.1%) to *Aedes*, 43 (10.4%) to *Armigeres*, 3 (0.7%) to *Mansonia* and 2 (0.5%) to *Toxorhynchites*. Among the 15 mosquito species identified, 131 mosquitoes (31.7%) were *An. sinensis*, 90 (21.8%) *Ae. albopictus*, 75 (18.2%) *Cx. quinquefasciatus*, 43 (10.4%) *Armigeres subalbatus*, 28 (6.8%) *Anopheles vagus*, 18 (4.4%) *Aedes lineatopennis* and 28 (6.8%) others (*Aedes vexans*, *Culex tritaeniorhynchus*, *Cx. gelidus*, *Cx. pipiens*, *Anopheles campestris*, *An. crawfordi*, *An. kochi*, *An. tessellatus*, *Mansonia uniformis* and *Toxorhynchites splendens*) (Table 1). Qiongzhong and Danzhou had the greatest mosquito diversity among the five study sites with eight mosquito species each, and Haikou had the lowest diversity with three species (Additional file 2: Table S2).

**Table 1:** Mosquito species composition and natural *Wolbachia* infection

Genus	Species^a^	N	N positive	Prevalence (%)
*Aedes*	***Ae. albopictus***	90	78	86.67
	*Ae. lineatopennis*	18	0	0
	*Ae. vexans*	4	0	0
*Armigeres*	***Ar. subalbatus***	43	16	37.21
*Culex*	***Cx. quinquefasciatus***	75	54	72.00
	*Cx. tritaeniorhynchus*	3	0	0
	***Cx. gelidus***	2	1	50.00
*Anopheles*	*An. sinensis*	131	0	0
	*An. campestris*	5	0	0
	*An. crawfordi*	2	0	0
	*An. kochi*	6	0	0
	*An. tessellatus*	1	0	0
	*An. vagus*	28	0	0
*Mansonia*	*Ma. uniformis*	3	0	0
*Toxorhynchites*	*T. splendens*	2	0	0
Total		413	149	36.08

^a^Species containing resident *Wolbachia* strains are in bold

All 413 mosquitoes were examined for *Wolbachia* infection based on the presence/absence of *wsp* genes. Four species, *Ae. albopictus*, *Cx. quinquefasciatus*, *Cx. gelidus* and *Ar. subalbatus*, were positive for *Wolbachia* infection, with an overall infection rate of 36.1% (149/413). *Wolbachia* infection rates varied substantially among infected species, with the lowest (37.2%) occurring in *Ar. subalbatus* and the highest (86.7%) in *Ae. albopictus* (Table 1). In *Ae. albopictus*, the majority of mosquitoes (64.1%, 50/78) were infected with both *wAlbA* and *wAlbB* strains of *Wolbachia*; mono-strain *wAlbA* and *wAlbB* infection rates were 21.1% and 10.0%, respectively (Additional file 3: Table S3). *Aedes albopictus* in Haikou had the highest infection rate (100%) and Lingao the lowest (65.0%). No *Wolbachia* infection was detected in any *Anopheles* mosquitoes.

**Fig. 2:**
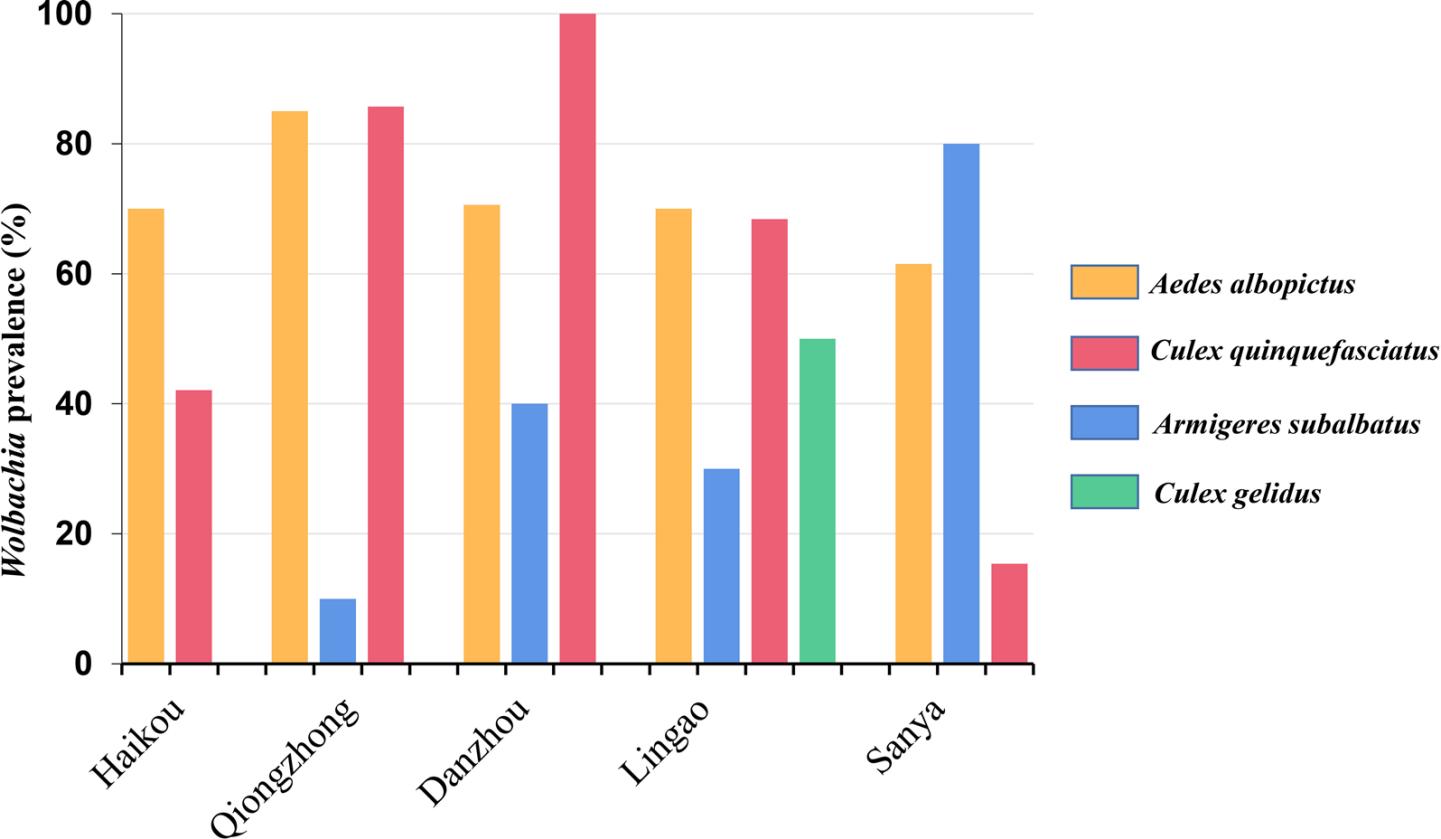
*Wolbachia* prevalence in Hainan Province based on PCR amplification of the *wsp* marker

The prevalence of *Wolbachia* infection also varied substantially among study sites (Fig. 2). Notably, not all mosquito species were found at all study sites, and sample sizes varied by species and study site (Additional file 3: Table S3); therefore, it is difficult to compare the composition of *Wolbachia* infections among different sites (Fig. 2).

### Genetic diversity and phylogenetic relationship of *Wolbachia* strains

A subset of 40 *Wolbachia*-infected female mosquitoes from the four species *Ae. albopictus*, *Ar. subalbatus, Cx. quinquefasciatus* and *Cx. gelidus* was used for DNA sequencing of the host *cox*1 gene and three *Wolbachia*-specific genes (*wsp*, *FtsZ* and *16S* rRNA). A total of 14 *cox*1 haplotypes were identified from the four mosquito species *Ae. albopictus* (5), *Ar. subalbatus* (6)*, Cx. quinquefasciatus* (2) and *Cx. gelidus* (1). A total of five *wsp* haplotypes, six *FtsZ* haplotypes and six *16S* rRNA haplotypes were detected from *Wolbachia* infections (Table 2). At least four *Wolbachia* strains (alb-wspH1/alb-FtsZH1/alb-16sH1, alb-wspH1/alb-FtsZH1/alb-16sH3, alb-wspH2/alb-FtsZH2/alb-16sH2 and alb-wspH1/alb-FtsZH3/alb-16sH1) were detected in *Ae. albopictus*, whereas two strains (sub-wspH1/sub-FtsZH1/sub-16sH1 and sub-wspH2/sub-FtsZH2/sub-16sH2) were found in *Ar. subalbatus* and one in each of *Cx. quinquefasciatus* (qui-wspH1/qui-FtsZH1/qui-16sH1) and *Cx. gelidus* (gel-wspH1/gel-FtsZH1/gel-16sH1)*.*

**Table 2:** Haplotypes of host mosquitoes and *Wolbachia* infections

Species	n	*cox1* haplotype	*wsp* haplotype	*FtsZ* haplotype	*16 s RNA* haplotype
*Aedes albopictus*	2	alb-cox1H1	alb-wspH1	alb-FtsZH1	alb-16sH1
	2	alb-cox1H1	alb-wspH1	alb-FtsZH1	alb-16sH3
	3	alb-cox1H1	alb-wspH1/H2^a^		
	3	alb-cox1H1	alb-wspH2	alb-FtsZH2	alb-16sH2
	1	alb-cox1H2	alb-wspH1/H2^a^		
	1	alb-cox1H3	alb-wspH1	alb-FtsZH1	alb-16sH1
	1	alb-cox1H4	alb-wspH1/H2^a^		
	1	alb-cox1H5	alb-wspH1	alb-FtsZH3	alb-16sH1
*Armigeres subalbatus*	4	sub-cox1H1	sub-wspH1	sub-FtsZH1	sub-16sH1
	2	sub-cox1H2	sub-wspH1	sub-FtsZH1	sub-16sH1
	1	sub-cox1H2	sub-wspH2	sub-FtsZH2	sub-16sH2
	2	sub-cox1H3	sub-wspH1	sub-FtsZH1	sub-16sH1
	2	sub-cox1H4	sub-wspH1	sub-FtsZH1	sub-16sH1
	1	sub-cox1H5	sub-wspH1	sub-FtsZH1	sub-16sH1
	1	sub-cox1H5	sub-wspH2	sub-FtsZH2	sub-16sH2
	1	sub-cox1H6	sub-wspH1	sub-FtsZH1	sub-16sH1
*Culex quinquefasciatus*	10	qui-cox1H1	qui-wspH1	qui-FtsZH1	qui-16sH1
	1	qui-cox1H2	qui-wspH1	qui-FtsZH1	qui-16sH1
*Culex gelidus*	1	gel-cox1H1	gel-wspH1	gel-FtsZH1	gel-16sH1
Total	40	14	5	6	6

^a^alb-wspH1/H2 represents mixed infections of wAlbA (alb-wspH2) and wAlbB (alb-wspH1) strains

**Fig. 3:**
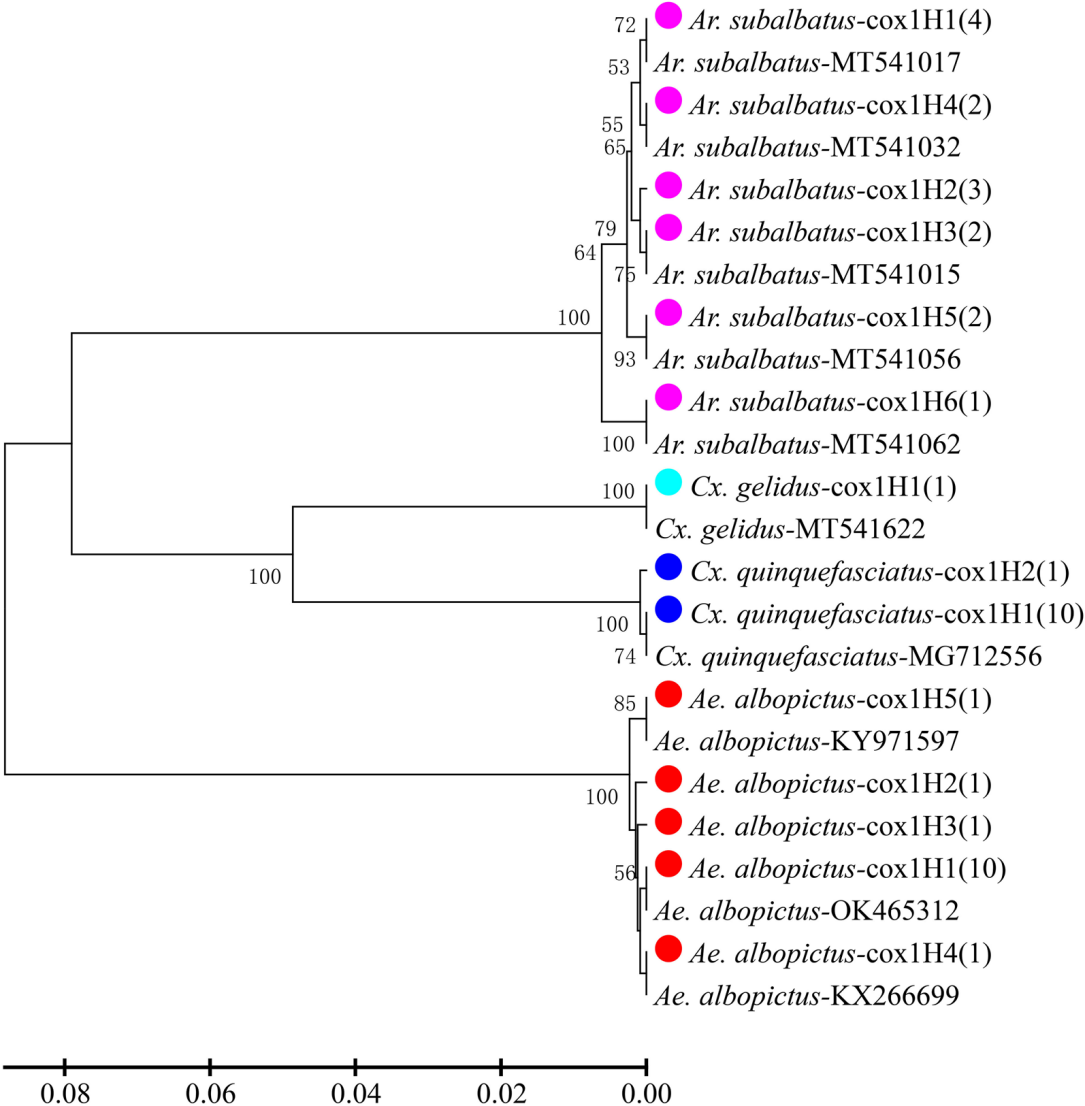
Phylogenetic tree analysis of *cox*1 haplotypes of different mosquito species collected in Hainan Province. Phylogenetic inference was performed using the UPGMA method. The percentage of replicate trees (> 50) in which the associated haplotypes clustered together in the bootstrap test (1000 replicates) is shown next to each branch. The evolutionary distances were computed using the Kimura two-parameter method; units are the number of base substitutions per site. Colored dots indicate haplotypes of different species identified in this study; numbers in parentheses indicate the abundance of each haplotype. Species name followed by GenBank accession number is provided for reference

**Fig. 4:**
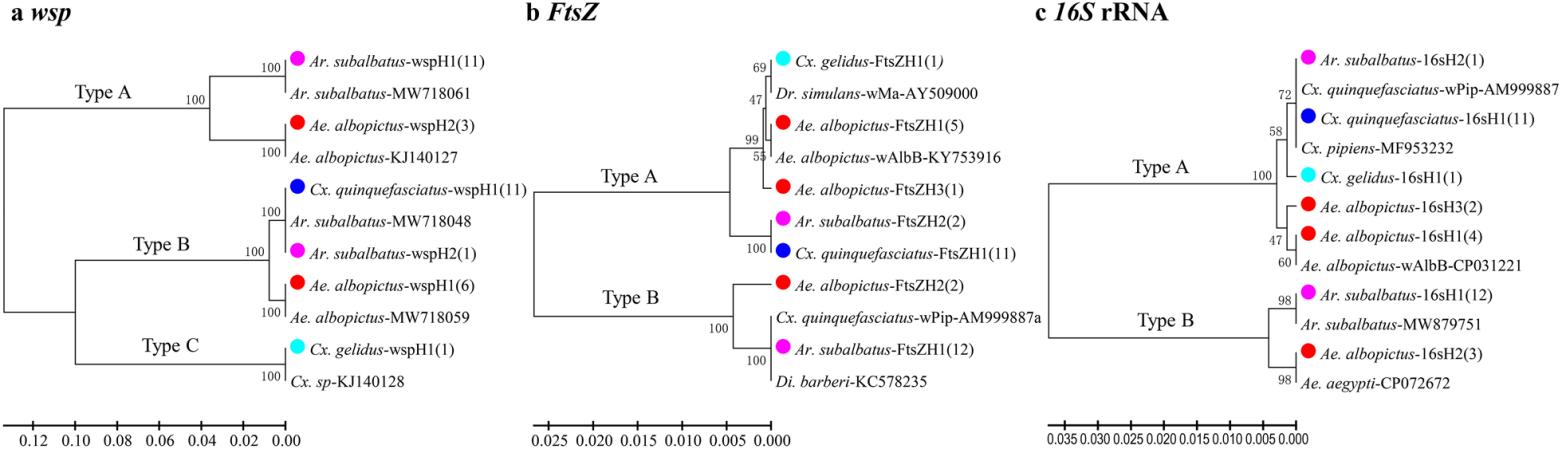
Phylogenetic tree analysis of the haplotypes of three *Wolbachia*-specific genes detected from mosquitoes in Hainan Province. **a** *wsp* gene sequences, **b** *FtsZ* gene sequences, **c** *16 s* rRNA gene sequences in *Wolbachia* strains. Phylogenetic inference was performed using the UPGMA method. The percentage of replicate trees (> 50) in which the associated haplotypes clustered together in the bootstrap test (1000 replicates) is shown next to each branch. The evolutionary distances were computed using the Kimura two-parameter method; units are the number of base substitutions per site. Colored dots indicate haplotypes of different species identified in this study; numbers in parentheses indicate the abundance of each haplotype. Species name followed by GenBank accession number is provided for reference

**Fig. 5:**
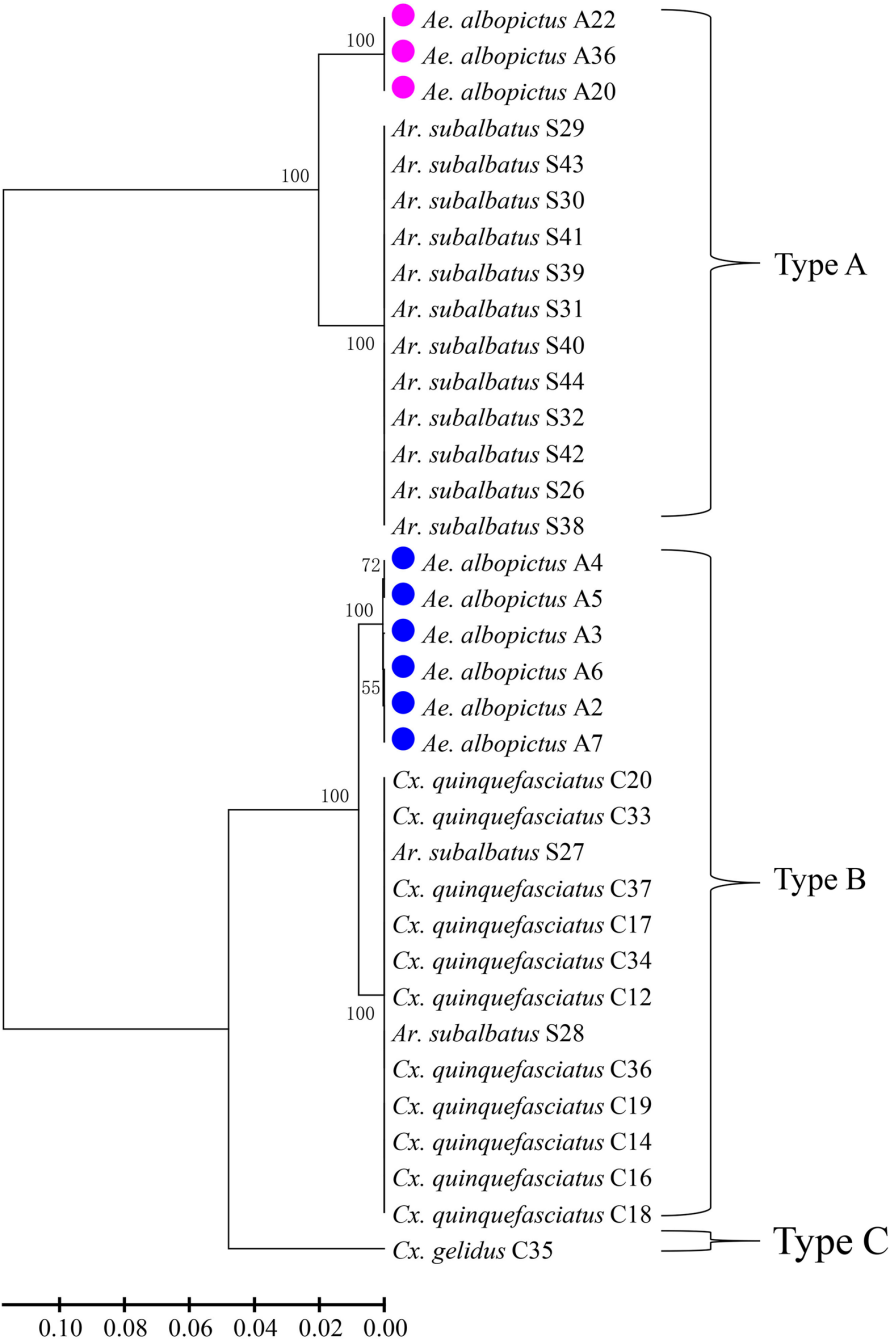
Multiple-loci sequence alignment analysis (MLSA) and phylogenetic inference of *Wolbachia* haplotypes resulting from combining three genes (*wsp, FtsZ* and 16 s rRNA) detected from mosquitoes in Hainan Province. Phylogenetic inference was performed using the UPGMA method. The percentage of replicate trees (> 50) in which the associated haplotypes clustered together in the bootstrap test (1000 replicates) is shown next to each branch. The evolutionary distances were computed using the Kimura two-parameter method; units are the number of base substitutions per site. Pink dots indicate individuals infected with wAlbA strain, while blue dots indicated individuals infected wAlbB strain, determined by both *wsp* gene alone and combining with *FtsZ* and 16 s rRNA sequencing in *Aedes albopictus*

Phylogenetic tree analysis of the mosquito *cox*1 gene showed clear separation into three clades, corresponding to the three genera (*Aedes, Armigeres* and *Culex*) (Fig. 3). *Wsp* sequences were classified into three groups of *Wolbachia* strains, corresponding to previously reported types A and B and a new group, namely type C (Fig. 4a). Both *FtsZ* (Fig. 4b) and *16S* rRNA (Fig. 4c) sequences were classified into two clades. When combining the three *Wolbachia* genes, the sequences of all mosquito specimens (single infection, *n* = 35) were grouped into three clades, corresponding to types A and B and type C (Fig. 5). The *Wolbachia* infections of *Ae. albopictus* were clearly classified into two clades (type A and type B) and which of *Cx. gelidus* was classified as type C, like those classifications based on *wsp* gene alone. The majority of *Wolbachia* infections in *Ar. subalbatus* were classified into type A, whereas two of them were grouped into type B. All the *Wolbachia* infections in *Cx. quinquefasciatus* were grouped into type B infections.

## Discussion

*Aedes* mosquitoes are responsible for 96 million dengue cases per year. Although the exact mechanisms are unclear, *Wolbachia*-modified *Aedes aegypti* mosquitoes prevent the spread of dengue virus through future bites [49, 50], which shows the potential of *Wolbachia* as a vector-suppression agent. In this study, we assessed the prevalence of *Wolbachia* in 15 female mosquito species collected from the field in Hainan, China, i.e. *Ae. albopictus*, *Ae. lineatopennis*, *Ae. vexans*, *Ar. subalbatus*, *Cx. quinquefasciatus*, *Cx. tritaeniorhynchus*, *Cx. gelidus*, *An. sinensis*, *An. campestris*, *An. crawfordi*, *An. kochi*, *An. tessellatus*, *An. vagus*, *Ma. uniformis* and *T. splendens*. *Wolbachia* was detected in four mosquito species. To our knowledge, this is the first comprehensive report to illustrate the presence and phylogeny of *Wolbachia* bacteria in natural mosquito populations in Hainan Province, including *Aedes*, *Culex*, *Anopheles*, *Armigeres*, *Mansonia* and *Toxorhynchites* mosquitoes, detected using *Wolbachia wsp*, *FtsZ* and *16S* rRNA PCR amplifications. As expected, the highest *Wolbachia* infection rate was in *Ae. albopictus* populations. Our results of total *Wolbachia* infection rate of 36.1% are comparable to those previously reported from neighboring countries such as Singapore (43.9%) [51], Thailand (61.5%) [52] and Malaysia (46.1) [44].

This study for the first time reported sequence variations of *Wolbachia* strains in* Cx. gelidus* mosquitoes. *Culex gelidus* is an emerging mosquito vector in India, Southeast Asia and Australia with the potential to transmit multiple viruses, including Japanese encephalitis virus (JEV), chikungunya (CKV), Ross River (RRV), Sindbis, Tembusu, West Nile (WNV), Kunjin and Murray Valley encephalitis viruses [53–55]. *Wolbachia* infections were previously reported in *Cx. gelidus* in central Thailand [56], while no infection was found in *Cx. gelidus* in Sri Lanka [57]. Due to the small number of mosquito specimens in this study, further studies are required to examine the distribution and phylogeny of *Wolbachia* strains in *Cx. gelidus*.

High genetic diversity of *Wolbachia* strains was found in *Ae. albopictus* and *Ar. subalbatus*, while low sequence variation was detected in *Cx. quinquefasciatus*. Most of the *Ae. albopictus* infections were a mixture of type A and type B *Wolbachia*, while *Cx. quinquefasciatus* was only infected with type B. Both type A and type B were detected in *Ar. subalbatus*, while a novel type C (*Cx.gelidus*-wspH1) was detected in *Cx. gelidus* mosquitoes. High rates of co-infection with type A and type B *Wolbachia* in *Ae. albopictus* have also been reported in other parts of China [27, 28], Argentina [58], Thailand [59] and Malaysia [60]. Co-infection with *wAlbA* and *wAlbB* was not observed in the natural population of *Cx. quinquefasciatus* in this study. In Indonesia, Shih et al. found that about 30% of *Cx. quinquefasciatus* were infected with group B *Wolbachia* and < 1% were infected with groups A and A&B [61]. A high proportion of *Ar. subalbatus* co-infected with *wAlbA* and *wAlbB* has been reported in Guangdong Province, China [62]. Studies found that *Ar. subalbatus* populations were infected with type A *Wolbachia* in Sri Lanka [57]. These regional variations in mosquito-*Wolbachia* interactions may represent an ongoing evolving process, or the infections may be occurring by chance or be associated with local environments. Further investigation is warranted.

In this study, we found no *Wolbachia*-infected *Anopheles* mosquitoes, including *An. sinensis*, *An. campestris*, *An. crawfordi*, *An. kochi*, *An. tessellatus* and *An. vagus*; this is similar to studies in Thailand [61], Italy [63], the USA [64] and Sri Lanka [57]. A few studies have found *Anopheles* mosquitoes infected with *Wolbachia*, such as in Tanzania [65], sub-Saharan Africa [66], Malaysia [44] and Burkina Faso [67]. Experiments on laboratory-reared *Anopheles* mosquitoes found that infection of *Wolbachia* in vector did affect the malaria parasite transmission. For example, Bian et al. found that the infection of *Anopheles stephensi* with *Wolbachia* wAlb B led to refractoriness to *Plasmodium* parasite infection [68]. Hughes et al. found that *Wolbachia* infections are virulent and inhibit the human malaria parasite *Plasmodium falciparum*'s development in *Anopheles gambiae* [69]. Shaw et al. found that *Wolbachia* infections in natural populations of *Anopheles coluzzii* negatively affected *Plasmodium* development [70]. It is possible that natural *Wolbachia* infection is variable in different areas; however, natural *Wolbachia* infection of wild *Anopheles* species is uncommon. Instances of *Wolbachia* infection in *Anopheles* mosquitoes should be further investigated, as previous studies suggest that the variability of strains found in some mosquito species (e.g. *Aedes*) may be due to environmental contamination rather than true *Wolbachia* infection [71]. For example, when collecting adult mosquitoes using CDC light traps, both *Culex* and *Anopheles* can be captured, and they are mixed (usually crashed) in the collection bag; contamination can occur at this stage—*Culex* harbors *Wolbachia* and *Anopheles* are contaminated.

We must note that the results from this study cannot be compared with experiments for DENV/ZIKV control in *Aedes* or *Culex* for WNV. First, the *Wolbachia* infection prevalence and strains are not comparable between them, because our data are from natural infection of *Wolbachia* in mosquitoes and the *Wolbachia* infections for DENV/ZIKV controls in Aedes are artificial (usually 100% prevalence with a uniform combination of strains) [14, 72]. Second, we do not know if the naturally occurring *Wolbachia* infection is enough to cause CI or blocking DENV/ZIKA/WNV transmission [73]. In addition, there are plenty of studies focusing on *Aedes* mosquitoes and *Aedes* transmitted viruses such as dengue, Zika and chikungunya viruses among others [14]. Only one *Wolbachia* strain is originally isolated from *Culex* mosquito against West Nile virus, i.e. *w*Pip from *Cx. quinquefasciatus* [73]. Although the two *Ae. aegypti* strains of *Wolbachia*, *w*Alb B and *w*MelPop, have been found to be good for *Culex* infections [74, 75], *w*MelPop is no longer being considered for field releases because of previous failures [26]. No specific *Wolbachia* strain has been found to block or reduce Japanese encephalitis virus (JEV) infection intensity [76]. Further investigation is desperately needed to study the *Wolbachia* infections in *Culex* mosquitoes transmitting WNV and JEV.

The three genetic markers (*16S* rRNA, *FtsZ* and *wsp* genes) have been widely used for characterization and classification of the insect endosymbiotic *Wolbachia* by single locus or multilocus sequence alignment (MLSA) analysis [45, 52, 77–79]. Eight supergroups have been designated (named A to H) primarily based on sequence data from the 16S rRNA, *FtsZ* and *wsp* genes [80, 81]. The majority of mosquito endosymbiotic *Wolbachia* strains belong to supergroups A and B [82]. In the current study, we observed similar results for the classifications of *Wolbachia* infection by using *wsp* gene alone or combining the three genes together, indicating a low or similar genetic diversity of *FtsZ* and *16S* rRNA genes compared to *wsp* genes. Further investigation may be needed using multilocus sequence typing (MLST) of the five genes (*FtsZ, fbpA, hcpA, coxA* and *gatB*) to reduce the confounding effect of genetic recombination [83]. MLST method may be more informative compared to sequencing a single marker, thus providing more accurate classifications of *Wolbachia* strains.

## Conclusions

This study demonstrated that *Wolbachia* infections were present in only a few mosquito species in Hainan Province, including the major dengue vector *Ae. albopictus*. Given the fact that *Wolbachia* can reduce the lifespan of some of its hosts, prevent certain pathogens from completing their life cycle and reduce the susceptibility of the host to certain pathogen infections, *Wolbachia* is being released on a small scale in many countries as an alternative vector control agent. The discovery of novel resident *Wolbachia* strains in local mosquito species in Hainan may also impact future attempts to expand *Wolbachia* biocontrol strategies for disease prevention. The long-term effects of introducing *Wolbachia* into new hosts and its effect on pathogen suppression should be thoroughly investigated.

## Supplementary Information

**Additional file 1: Table S1.** Primers for amplification and sequencing.

**Additional file 2: Table S2.** Mosquito diversity among the five study areas in Hainan Province, China.

**Additional file 3: Table S3.** Infection status of *Wolbachia* based on PCR results of field-collected *Aedes albopictus* adults.

## Data Availability

The datasets used and/or analyzed during this study are included in this published article. Nucleotide sequences generated in this study have been submitted to GenBank (accession nos. OP279050-OP279063, OP367764-OP367777, OP363894-OP363900, OP393144-OP393149 and OP426265-OP426271).
