# Supplementary material for: Characterizing the Wolbachia infection in field-collected Culicidae mosquitoes from Hainan Province, China
Source: Parasit Vectors. 2023 Apr 14;16:128. doi: 10.1186/s13071-023-05719-y (PMC10103416; doi:10.1186/s13071-023-05719-y)
Supplement: Supplementary file 1 — Additional file 1: Table S1. Primers for amplification and sequencing. [file 13071_2023_5719_MOESM1_ESM.docx]

Table S1 Primers for amplification and sequencing

| Gene/Species | Primer | Sequence（5'-3'） | Annealing T（℃） |
| --- | --- | --- | --- |
| wAlbA-*wsp* | WAF | CCAGCAGATACTATTGCG | 55 |
|  | WAR | AAAAATTAAACGCTACTCCA |  |
| wAlbB-*wsp* | WBF | AAGGAACCGAAGTTCATG | 55 |
|  | WBR | AAAAATTAAACGCTACTCCA |  |
| *wsp* | 81F | TGGTCCAATAAGTGATGAAGAAAC | 50 |
|  | 691R | AAAAATTAAACGCTACTCCA |  |
| *FtsZ* | *ftsZ*-F | TACTGACTGTTGGAGTTGTAACTAAGCCGT | 60 |
|  | *ftsZ*-R | TGCCAGTTGCAAGAACAGAAACTCTAACTC |  |
| *16S* rDNA | W-Specf | CATACCTATTCGAAGGGATAG | 60 |
|  | W-Specr | AGCTTCGAG TGAAACCAATTC |  |
|  | 16SNF | GAAGGGATAGGGTCGGTTCG | 60 |
|  | 16SNR | CAATTCCCATGGCGTGACG |  |
| COI | COI-F | TTTACAATTTATCGCCTAAACTTC | 55 |
|  | COI-R | CATTGCACTAATCTGCCATA |  |
| *Aedes albopictus* | Abl-F | CACCCGTGTATGTGCGATATTA | 59 |
|  | Abl-R | TTGGTCGTTCGGTGGTAAAG |  |
| *Culex quinquefasciatus* | Culex Q-F | CCTTCTTGAATGGCTGTGGCA | 55 |
|  | Culex Q-R | TGGAGCCTCCTCTTCACGG |  |
| *Anopheles sinensis* | *An. sinensis*-F | TGTGAACTGCAGGACACATGAA | 57 |
|  | *An. sinensis*-R | AGGGTCAAGGCATACAGAAGGC |  |

Note: Primers cox1-F, 81F, ftsZ-F,16SNF were used for sequencing

Abbreviation: T, temperature
