# Supplementary material for: Characterizing the Wolbachia infection in field-collected Culicidae mosquitoes from Hainan Province, China
Source: Parasit Vectors. 2023 Apr 14;16:128. doi: 10.1186/s13071-023-05719-y (PMC10103416; doi:10.1186/s13071-023-05719-y)
Supplement: Supplementary file 2 — Additional file 2: Table S2. Mosquito diversity among the five study areas in Hainan Province, China. [file 13071_2023_5719_MOESM2_ESM.docx]

Table S2. Mosquito diversity among the five study areas in Hainan Province, China

| Study area | N | Species |
| --- | --- | --- |
| Haikou | 3 | *Aedes albopictus* |
|  |  | *Armigeres subalbatus* |
|  |  | *Culex quinquefasciatus* |
| Qiongzhong | 8 | *Aedes albopictus* |
|  |  | *Armigeres subalbatus* |
|  |  | *Culex quinquefasciatus* |
|  |  | *Anopheles sinensis* |
|  |  | *Anopheles campestris* |
|  |  | *Anopheles crawfordi* |
|  |  | *Anopheles kochi* |
|  |  | *Anopheles tessellatus* |
| Danzhou | 8 | *Aedes albopictus* |
|  |  | *Armigeres subalbatus* |
|  |  | *Culex quinquefasciatus* |
|  |  | *Culex gelidus* |
|  |  | *Mansonia uniformis* |
|  |  | *Anopheles sinensis* |
|  |  | *Anopheles campestris* |
|  |  | *Anopheles vagus* |
| Lingao | 7 | *Aedes albopictus* |
|  |  | *Armigeres subalbatus* |
|  |  | *Culex quinquefasciatus* |
|  |  | *Aedes vexans* |
|  |  | *Aedes lineatopennis* |
|  |  | *Culex tritaeniorhynchus* |
|  |  | *Toxorhynchites splendens* |
| Sanya | 4 | *Aedes albopictus* |
|  |  | *Armigeres subalbatus* |
|  |  | *Culex quinquefasciatus* |
|  |  | *Anopheles sinensis* |

Note: N represents number of mosquito species.
