# Supplementary material for: Characterizing the Wolbachia infection in field-collected Culicidae mosquitoes from Hainan Province, China
Source: Parasit Vectors. 2023 Apr 14;16:128. doi: 10.1186/s13071-023-05719-y (PMC10103416; doi:10.1186/s13071-023-05719-y)
Supplement: Supplementary file 3 — Additional file 3: Table S3. Infection status of Wolbachia based on PCR results of field-collected Aedes albopictus adults. [file 13071_2023_5719_MOESM3_ESM.docx]

Table S3. Infection status of *Wolbachia* based on PCR results of field-collected *Ae. albopictus* adults

| Study areas | Total | No. of infected (%) | | | |
| --- | --- | --- | --- | --- | --- |
|  |  | Single A | Single B | A and B | W^+^ |
| Haikou | 20 | 2 (10.0) | 0 (0) | 18 (90.0) | 20 (100.0) |
| Qiongzhong | 20 | 3 (15.0) | 5 (25.0) | 11 (55.0) | 19 (95.0) |
| Danzhou | 17 | 6 (35.3) | 1 (5.9) | 7 (41.2) | 14 (82.4) |
| Lingao | 20 | 4 (20.0) | 0 (0) | 9 (45.0) | 13 (65.0) |
| Sanya | 13 | 4 (30.8) | 3 (23.1) | 5 (38.5) | 12 (92.3) |
| Total | 90 | 19 (21.1) | 9 (10.0) | 50 (55.6) | 78 (86.7) |

Note: W^+^ reprsents the positive rate of *Wolbachia* in *Ae. albopictus*
